# Dr. William Harland Peake

**Published:** 1910-06-11

**Authors:** 


The death is announced of Dr. William Harland Peake at the age of 40. He studied at Guy's and Durham University, taking his diploma in 1895, his M.B. and B.S. in that year also, and in 1897 became an M.D. of his University. His appointments included those of junior and senior clinical assistant to the Throat Hospital, Golden Square, to the out-patient department of the Evelina Children's Hospital, and to the Royal Westminster Ophthalmic Hospital. He also contributed once or twice to the medical papers.

